# Socioeconomic and Environmental Factors Associated With Increased Alcohol Purchase and Consumption in 38 Countries During the Covid-19 Pandemic

**DOI:** 10.3389/fpsyt.2021.802037

**Published:** 2022-01-14

**Authors:** Zaheer Kyaw Hla, Rodrigo Ramalho, Lauranna Teunissen, Isabelle Cuykx, Paulien Decorte, Sara Pabian, Kathleen Van Royen, Charlotte De Backer, Sarah Gerritsen

**Affiliations:** ^1^Social and Community Health, School of Population Health, University of Auckland, Auckland, New Zealand; ^2^Department of Communication Sciences, Faculty of Social Sciences, University of Antwerp, Antwerp, Belgium; ^3^Department of Communication and Cognition, Tilburg University, Tilburg, Netherlands; ^4^Department of Primary and Interdisciplinary Care, University of Antwerp, Antwerp, Belgium

**Keywords:** alcohol consumption/use, drinking, Covid-19 pandemic, risk factors, public health, crisis, alcohol purchase, mental health

## Abstract

**Aims:**

To explore changes in alcohol purchase and consumption during the first few months of the Covid-19 pandemic, and assess associations between increased alcohol purchase/use and socioeconomic and environmental factors.

**Design:**

Secondary data from a cross-sectional online survey conducted from 17 April to 25 June 2020.

**Setting:**

Thirty-eight countries from all continents of the world.

**Participants:**

A total of 37,206 adults (mean age:36.7, SD:14.8, 77% female) reporting alcohol purchasing and drinking habit before and during the pandemic.

**Measurements:**

Changes in alcohol stock-up and frequency of alcohol use during the pandemic and increased alcohol stock-up and use were stratified by gender, age, education, household structure, working status, income loss, psychological distress, and country based on alcohol consumption per capita. The associations between increased alcohol stock-up/use and living with children, working from home, income loss and distress were examined using multivariate logistic regression, controlling for demographic factors.

**Findings:**

The majority of respondents reported no change in their alcohol purchasing and drinking habits during the early pandemic period. Increased drinking was reported by 20.2% of respondents, while 17.6% reported decreased alcohol use. More than half (53.3%) of respondents experienced psychological distress, with one in five (20.7%) having severe distress. Female gender, being aged under 50, higher educational attainment, living with children, working from home, and psychological distress were all independently associated with increased alcohol drinking during lockdown. Limitations of the study were the non-representative sample, the data collection early in the pandemic, and the non-standard measurement of alcohol consumption.

**Conclusion:**

Increased psychological distress among people during the early pandemic period, resulted in increased alcohol consumption, especially among women with children working from home during lockdown.

## Introduction

The Covid-19 pandemic has increased the likelihood of people experiencing income or job loss, losing loved ones, being stuck at home or in a foreign country alone, and uncertainty about the future (1). People often resort to harmful coping mechanisms during an acute stressful period, notably by increasing alcohol and other drug use (2, 3). For example, a study with Hong Kong residents found that regular drinkers had increased their alcohol use one year after the 2003-SARS pandemic (4). Unfortunately, prioritisation of health resources toward pandemic-related services has resulted in diminished support for those suffering from alcohol-related harms, including alcohol-withdrawal symptoms (5, 6).

Some countries like South Africa, Thailand, and India have had limited alcohol availability during lockdowns implemented during the pandemic (7–9). However, alcohol sales were promoted extensively through online marketing and home deliveries, especially in Western countries (6, 10, 11), where there is already a high quantity of alcohol consumed (12). This may have resulted in an increased prevalence of home drinking in these countries during lockdowns. Public health experts have warned against the risks of this situation, which include a higher onset of alcohol dependence, intimate partner violence, and negative emotional and physical health impacts on children (10, 13). Alcohol misuse also increases susceptibility to Covid-19 infection through changes in the respiratory system (14) and impairing the body's immune system (15, 16).

It is essential to pay attention to the pandemic's impact on alcohol use patterns. While some authors have reported increased alcohol use during the pandemic, others have shown a reverse trend (17–20). A systematic review reported that most studies conducted on this topic had not found any overall changes in alcohol use (21). Individual differences between specific sub-groups of people have likely remained hidden when presented as part of a homogeneous larger population. Further research is needed to develop a better understanding of the risk factors associated with increased alcohol use. This would allow appropriate healthcare and support to be provided to protect vulnerable groups from long-term health consequences.

This study aimed to use data from an international survey conducted in 38 countries during the early period of the Covid-19 pandemic to assess changes in alcohol purchase and consumption, and determine factors associated with increased stock-up and use of alcohol during lockdowns.

## Methods

### Data

This study used secondary data collected in an international cross-sectional online survey named the Corona Cooking Survey (22). Data were collected from 38 countries, mostly in native languages, from 17 April to 25 June 2020. The self-administered questionnaire was designed in Qualtrics by researchers at the University of Antwerp. This university granted initial ethical approval for the study (ref no: SHW_19_44), although each country also received approval from its respective ethical body. Participants (18+ years) were recruited via convenience/snowball sampling. The survey was promoted through various methods, e.g., press releases, professional networks, targeted adverts on social media, and the donation of one Euro per completed questionnaire (maximum 3,000 Euro) to the Global Food Bank Network. Informed consent was obtained in a separate form with information about the survey and participant's rights. The number of participants from each country varied from fewer than 200 to more than 5,000 at the end of the completed survey (see Supporting information Supplementary Table S1 for the country list with gender-wise participant numbers). The study protocol can be accessed *via* https://osf.io/nz9xf/files/. The link also offers access to the survey questions and the literature supporting their inclusion in the survey.

### Measures

Changes in alcohol use were captured by two separate questions about individual drinking frequency before and during lockdown in a seven-point frequency response scale, ranging from never and less than once per week to twice or more per day. The survey asked how often the participant consumed at least a portion^1^ (a glass) of alcoholic beverages. Alcohol stock-up^2^ information during the pandemic was collected using a slightly different seven-point response scale, containing options from “1 = a lot less than usual” to “7 = a lot more than usual” at two opposite ends. Participants were asked to compare their stocking-up pattern of alcoholic drinks with their regular stock-up pattern before the pandemic. The alcohol stock-up question was not responded to by about 50% of the participants partly because the alcohol stock-up question was not included in Arab countries where alcohol is prohibited or not widely available. The outcome variables of change in alcohol use were created by comparing the proportion of people with different frequencies of alcohol use before and during lockdown. A categorical variable was created to simplify increase or decrease with three groups (never, less than daily, and daily). A similar variable for alcohol stock-up was created by combining its frequencies into three categories–less, similar, and more. Binary categorical variables for increased drinking frequency and increased alcohol stock-up were also created for use in the multivariate analysis.

Table 1 offers some details about the 38 countries included in the study, i.e., income status, minimum alcohol purchase age, and the stringency index at the time of the survey. The stringency index offers a indication of the government's pandemic response in a scale from 0 to 100, with 100 meaning strictest lockdown measures (23). However, for the purpose of this study, these countries have been grouped according to the average alcohol consumption per capita (APC) (Table 2). The APC offers a useful insight into the trends of alcohol consumption of the adult population of a country (24). This resulted in four categories, based on the latest WHO global alcohol consumption report (25). Low and medium countries were combined for data analysis, as were the high and very high consumption countries, due to small numbers of participants in the medium and high consumption groups.

**Table 1:** Countries' income status, alcohol regulation and stringency index.

**Country**	**Income Status^**a**^**	**MPA^**b**^**	**Stringency Index^**c**^**	
			17-Apr-20	25-Jun-20
Australia	High	Age 18 and above	69.44	52.31
Austria	High	Age 16/18 and above	77.78	50
Bahrain	High	Age 21+ Non-Muslim	75	75
Belgium	High	Age 16/18 and above	81.48	51.58
Brazil	Upper-middle	Age 18 and above	74.54	77.31
Canada	High	Age 18/19 and above	72.69	68.89
Chile	High	Age 18 and above	73.15	78.24
China	Upper-middle	No age restriction	56.94	78.24
Denmark	High	Age 16/18 and above	68.52	57.41
Ecuador	Upper-middle	Age 18 and above	93.52	79.63
Egypt	Low-middle	Age 21 and above	84.26	71.3
Finland	High	Age 18/20 and above	68.52	35.19
France	High	Age 18 and above	87.96	51.85
Germany	High	Age 16/18 and above	76.85	63.43
Greece	High	Age 18 and above	84.26	50
Ireland	High	Age 18 and above	90.74	72.22
Italy	High	Age 18 and above	93.52	67.59
Japan	High	Age 20 and above	47.22	25.93
Jordan	Upper-middle	Age 18 and above	100	48.15
Kuwait	High	Total ban	93.52	83.8
Lebanon	Upper-middle	Age 18 and above	85.19	74.07
Mexico	Upper-middle	Age 18 and above	82.41	70.83
Netherlands	High	Age 18 and above	78.7	59.26
New Zealand	High	Age 18 and above	96.3	22.22
Oman	High	Age 21 and above	92.59	87.96
Palestine	Low-middle	Age 18 and above	96.3	80.56
Peru	Upper-middle	Age 18 and above	94.44	89.81
Poland	High	Age 18 and above	87.04	50.93
Qatar	High	Non-Muslim adults	86.11	80.56
Romania	Upper-middle	Age 18 and above	87.04	41.67
Saudi Arabia	High	Total ban	91.67	71.3
Singapore	High	Age 18 and above	76.85	50.93
South Africa	Upper-middle	Age 18 and above	87.96	76.85
Spain	High	Age 18 and above	85.19	41.2
Uganda	Low	Age 18 and above	93.52	87.04
United Arab Emirates	High	Need a licence or permit to buy	87.04	72.22
United Kingdom	High	Age 18 and above	79.63	71.3
United States	High	Age 21 and above	72.69	68.98

a *World Bank. World Bank Country and Lending Groups [Internet].: The World Bank; 2021 [cited 17 November 2021]*.

b *MPA–Minimum Purchase Age. Data retrieved from the World Health Organisation. Global status report on alcohol and health 2018. World Health Organisation; 2019*.

c *University of Oxford. Covid-19 Government Response Tracker [Internet].: University of Oxford; 2021 [cited 17 November 2021]*.

**Table 2:** Four categories of participating countries based on APC status.

**Countries**	**APC Category**
Arab countries, Ecuador, Singapore	Low (<5L per capita)
China, Mexico, Peru	Medium (5–7.4 L per capita)
Brazil, Chile, Canada, Japan, Italy, Netherlands, South Africa, Uganda, USA	High (7.5–9.9 L per capita)
Austria, Australia, Belgium, Denmark, Finland, France, Germany, Greece, Ireland, New Zealand, Poland, Romania, Spain, UK	Very high (≥10 L per capita)

Psychological distress was measured using a modified version of the Kessler (K6) scale that asks participants about six different feelings with seven-point frequency response options (1 = never to 7 = all the time) over the last 30 days (26). This scale's internal consistency in the sample was high (α = 0.88). The K6 responses were modified by grouping the seven-point scale into a five-point scale (0 to 4) for each item, so that the cut-off score for mental distress can be standardised according to the literature (27). Recoding was done as follows: never = 0, very rarely or rarely = 1, sometimes = 2, frequently or very frequently = 3, and all the time = 4. A total score of 13 and greater indicated severe distress, a score between 7 and 13 was moderate, and a score of equal to seven and less was considered normal (27). Socio-demographic information, including household structure and employment status, was also collected. Economic consequences due to the pandemic were collected with an income loss question that was asked with a binary response (yes or no).

### Statistical Analysis

Data were analysed using the statistical software Stata 16 (StataCorp, Texas). First, descriptive data analysis techniques were used to assess changes in pattern of alcohol stock-up and use, the prevalence of psychological distress during lockdown, and participants' socioeconomic and demographic profile. Prtest (*t*-test for proportions) was used to observe significant of changes in alcohol stock-up and use before and during lockdown or pandemic. Univariate analysis was used to observe associations between independent variables and outcome variables. Statistical significance was assessed with Pearson's chi square test with a significance threshold set at *p* = 0.05. The second stage of data analysis was done by creating multivariate models for increased alcohol stock-up and use (separately) with predictor variables (household structure, employment status, lost income, and psychological distress) one at a time with sociodemographic factors (gender, age, education, and country group based on APC) included in every model. A final model for each alcohol variable (increased alcohol stock-up and use) was developed by adding all factors to the model, taking psychological distress as the main predictor variable. Univariate logistic analysis was also used to observe the effect of increased alcohol stock-up on increased alcohol use.

## Results

Although 81,486 people started the survey, the survey closed with 38,666 completed responses, of which 37,206^3^ participants were left for the analysis after data cleaning. Some (1,460) respondents were removed from the data as part of data cleaning process (one who did not fill gender, two with invalid age (>99 year), 128 with diverse gender, 479 with invalid resident country, 849 who did not provide their country of residence and one who did not respond to alcohol use question). More than a quarter (26.2%) of participants stocked up a larger quantity of alcohol during lockdown compared to before the pandemic. About one in eight (13.3%) stocked up less alcohol during the pandemic and more than half (60.5%) bought the same amount as they did before the pandemic. One in three (32.6%) reported not drinking alcohol before lockdown, which increased to 37.4% during lockdown. Over half (51.4%) of the respondents were less than daily drinkers before lockdown, which decreased to 42.8% during lockdown. However, the proportion of participants who drank daily increased from 16.1% before lockdown to 19.8% during lockdown.

**Figure 1:**
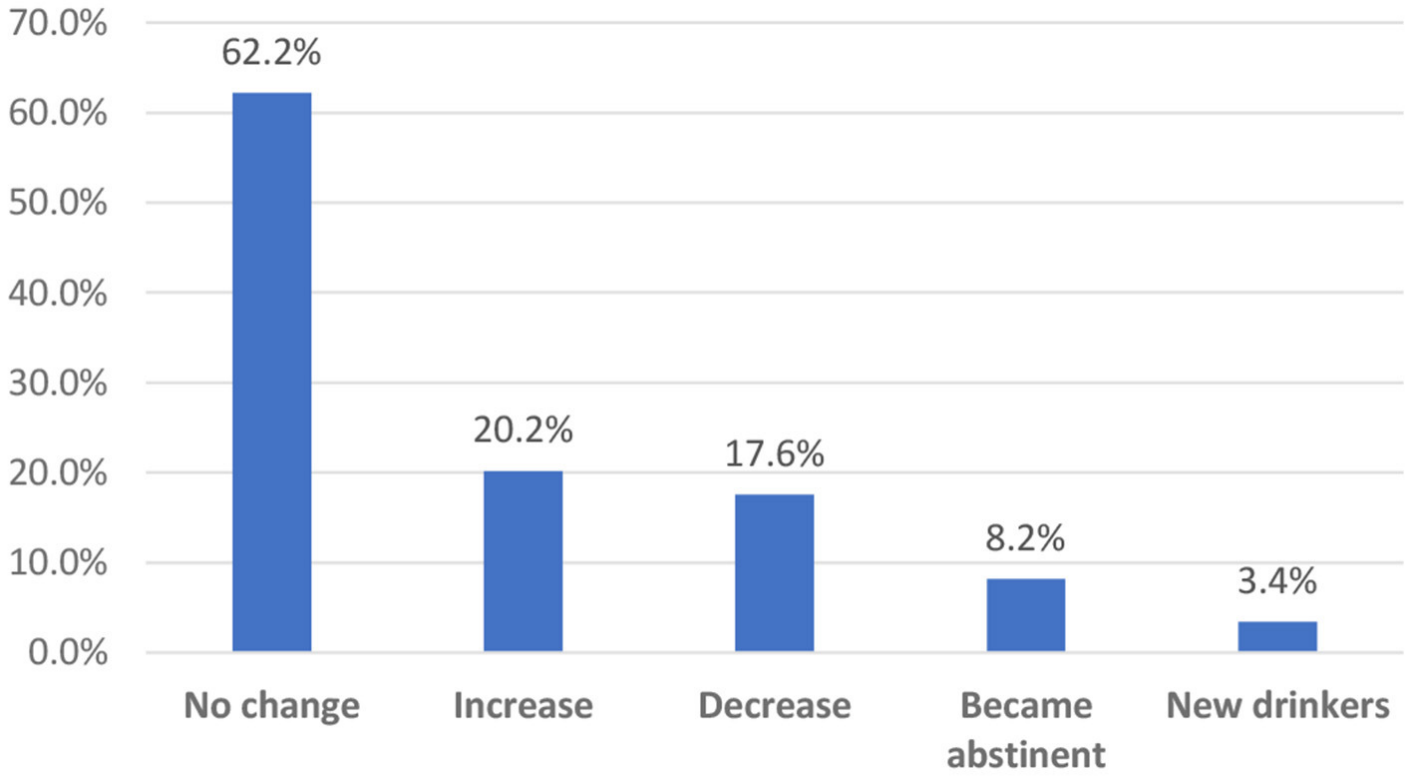
Proportion of changes in alcohol use due to lockdown (*n* = 37,206).

Figure 1 shows the proportion of changes in alcohol use due to lockdown. The majority of participants (62.2%) reported no changes in their drinking patterns during the lockdown. The 20.2% who increased their drinking frequency comprised about 10% of the participants who increased to daily and 10% who increased to less than daily. Likewise, the 17.6% of participants who decreased their alcohol use were made up of almost 5% who reported a decrease from daily and 13% from less than daily drinking. The proportions for the abstinent people and new users were also reflected in the decreased and increased proportions, respectively.

### Socioeconomic and Environmental Factors Associated With Increased Alcohol Purchase and Use

Sample characteristics along with the association between different factors and outcome variables are shown in Table 2. Stocking-up on alcohol was higher among younger age groups (18–25 years) compared to those aged above 49, graduates and postgraduates compared to those who studied up to high school, those working at workplace and from home compared to those who were unemployed, those who reported income loss compared to those who did not report income loss, and those who experienced moderate and severe psychological distress compared to those who did not report distress. Those who were living alone reported less alcohol stock-up compared to those who were living only with adults.

Increased alcohol use was found among; females compared to males, younger age groups compared to those aged above 49, graduates and postgraduates compared to those who studied up to high school, those from countries with higher APC compared to those from countries with low and medium APC, those who were living with children compared to those who were living only with adults, those working at workplace and from home compared to those who were unemployed, and those who experienced moderate and severe distress compared to those who did not reported distress (Table 3).

**Table 3:** Descriptive statistics of sample characteristics and associations with increased alcohol stock-up and use.

**Variable**	**Increased alcohol stock-up**		**Increased alcohol use**	
	**No. (%)**	**OR (95% CI)**	**No. (%)**	**OR (95% CI)**
**Total**	16,288 (100)	_	37,206 (100)	_
Gender				
Male	990 (23.2)	Reference	8,539 (23.0)	Reference
Female	3,274 (76.8)	1.06 (0.97–1.15)	28,667 (77.0)	**1.16 (1.09–1.24)**
Age group				
18–25	620 (14.5)	**1.53 (1.37–1.72)**	11,751 (31.6)	**1.23 (1.14–1.33)**
26–49	2,623 (61.5)	**1.68 (1.55–1.83)**	17,338 (46.6)	**1.5 (1.4–1.61)**
>49	1,021 (24.0)	Reference	8,117 (21.8)	Reference
Mean (SD) age	_	_	36.7 (14.8)	_
Education				
High school	890 (20.9)	Reference	10,144 (27.3)	Reference
Bachelor	1,649 (38.7)	**1.33 (1.21–1.46)**	16,722 (28)	**1.23 (1.16–1.31)**
Postgraduate	1,725 (40.5)	**1.38 (1.26–1.51)**	10,334 (27.8)	**1.38 (1.29–1.48)**
Country-based on APC				
Low and medium (<7.5L)	376 (8.8)	Reference	16,383 (29)	Reference
High and very high (≥ 7.5L)	3,888 (91.2)	1.01 (0.9–1.15)	20,823 (30)	**1.29 (1.23–1.36)**
Household structure				
Adult only	2,147 (50.4)	Reference	16,053 (43.2)	Reference
Includes child	1,531 (35.9)	1.08 (1–1.17)	18,197(48.9)	**1.13 (1.07–1.19)**
Alone	586 (13.7)	**0.88 (0.79–0.97)**	2,956 (7.9)	0.92 (0.83–1.02)
Employment status				
Working at workplace	582 (13.7)	**1.14 (1.01–1.29)**	3,530 (9.5)	**1.13 (1.03–1.23)**
Working from home	2,560 (60.1)	**1.67 (1.52–1.82)**	13,962 (37.5)	**1.52 (1.43–1.63)**
Student (not working)	298 (7.0)	1.14 (0.98–1.33)	7,535 (20.3)	1.05 (0.97–1.14)
Unemployed	824 (19.3)	Reference	10,210 (27.4)	Reference
Income loss				
No	2,957 (69.3)	Reference	24,813 (66.7)	Reference
Yes	1,307 (30.7)	**1.18 (1.09–1.27)**	12,392 (33.3)	1.01 (0.96–1.07)
Kessler mental stress score (out of 24)				
Normal (≤ 7)	1,935 (45.4)	Reference	17,383 (46.7)	Reference
Moderate (8–12)	1,461 (34.3)	**1.48 (1.37–1.6)**	12,113 (32.6)	**1.23 (1.16–1.3)**
Severe (≥ 13)	868 (20.4)	**1.93 (1.75–2.13)**	7,710 (20.7)	**1.3 (1.23–1.4)**

*Bold font indicates statistical significance in the univariate analysis*.

Multivariate analysis showed living alone was negatively associated with increased alcohol stock-up compared to those living only with adults, after adjusting for the demographic factors (Table 4). Working from home increased the probability of more alcohol stock-up by 1.43 times compared to those who were unemployed, after adjusting for the demographic factors. Income loss was also associated with increased alcohol stock-up, after adjusting for the demographic factors. The effect of distress on increased alcohol stock-up increased as severity of distress level raised, after adjusting for the demographic factors.

**Table 4:** Results of multivariate regressions on increased alcohol stock-up and use.

**Predictor variable**	**Increased alcohol stock-up (*****n*** **=** **4,264)**			**Increased alcohol use (*****n*** **=** **7,502)**		
	**Odds Ratio**	**95% CI**	***p*** **-value**	**Odds Ratio**	**95% CI**	***p*** **-value**
Model 1: Household structure (reference: only adults)						
Includes child	0.96	0.88–1.04	0.271	1.17	1.10–1.24	<0.001
Alone	0.86	0.78–0.96	0.006	0.87	0.79–0.97	0.01
Model 2: Working status (reference: unemployed)						
Working at workplace	1	0.88–1.13	0.972	1.05	0.97–1.15	0.247
Working from home	1.43	1.30–1.57	<0.001	1.34	1.25–1.44	<0.001
Student (not working)	0.86	0.72–1.02	0.078	0.94	0.86–1.03	0.206
Model 3: Income loss (reference: no income loss)						
Yes	1.18	1.09–1.28	<0.001	1.06	1.01–1.12	0.03
Model 4: Psychological distress (reference: normal)						
Moderate	1.41	1.30–1.53	<0.001	1.23	1.16–1.30	<0.001
Severe	1.89	1.71–2.09	<0.001	1.37	1.28–1.47	<0.001

*Each model was adjusted for gender, age, education and APC-based countries group*.

Those whose households included at least one child increased drinking by 1.17 times compared to those living only with adults, after adjusting for the demographic factors (Table 4). In contrast, living alone seemed to be protective for increased alcohol use compared to those living with other adults. People working from home increased alcohol use by 1.34 times compared to those who were unemployed, after adjusting for the demographic factors. Losing income resulted in increased alcohol use compared to those who did not experience income loss, although the effect size is very small. The effect of psychological distress on increased alcohol use demonstrated an exposure-response relationship, increasing the risk of frequent drinking as the severity of distress increased during lockdowns.

In the final (complete) model, increased alcohol stock-up during the pandemic was found among those with psychological distress, working from home, aged <50 years, with higher education attainment, and those from higher APC countries (see Supporting information Supplementary Table S2 for the detailed multivariate results). In contrast, living alone and losing income were protective against increased alcohol stock-up. Living with children and female gender had no association with increased alcohol stock-up in this final model. The effect of severe distress on increased alcohol use became stronger in the final (combined) model, along with the effects of living with children and working from home, which also remained statistically significant (see Supporting information Supplementary Table S3 for the detailed multivariate results). The effects of demographic factors on increased alcohol use also remained significant in this final model. The income loss variable was dropped from the final model for increased alcohol use because its effect became insignificant.

A strong correlation was found between increased alcohol stock-up and increased alcohol use during the pandemic. The univariate logistic analysis showed risk of increased alcohol use was five times higher (95% CI = 4.70, 5.51) in those who increased alcohol stock-up during the pandemic.

## Discussion

The present study explored the factors associated with increased alcohol stock-up and consumption during the early period of the Covid-19 pandemic. This is the first study to report changes in stocking up on alcohol during the pandemic. In the present study, the proportion of people who increased their alcohol drinking during the early phase of the pandemic was larger than the proportion of people who decreased it. Middle-aged, educated women, living with children and working from home appeared to be a high-risk group for increased alcohol use during lockdown. They were more likely to experience psychological distress and increase their drinking frequency. Also, more than a quarter of people stocked up on alcohol during the early pandemic period, and those who stockpiled alcohol were more likely to increase the frequency and quantity of alcohol drinking. Finally, the increased alcohol use during the pandemic was more likely to occur in countries with a pre-pandemic higher average quantity of APC, countries which are mostly those members of the Organisation for Economic Cooperation and Development (OECD).

According to the latest World Bank classification, the majority of participating countries were in the upper-middle- and high-income categories (see details in Table 1). Generally, countries' income levels correlate with alcohol consumption except in Arab countries where alcohol purchase and drinking are strictly regulated. Several Arab countries, like Qatar and UAE, with a large population of expatriates, allow eligible adults to purchase and drink alcohol. There were only two Muslim countries where alcohol use is banned (Table 1). Regarding the governments' pandemic response stringency index, where 100 mean strictest lockdown measures, the average indices of participating countries were about 82 and 64 at the beginning and end of the survey period, respectively. This indicates an overall similar stringency index across countries. Plus, it is also worth considering that the majority of alcohol drinking happened at home during the pandemic.

The finding that more than a quarter of the respondents increased alcohol stock-up is higher than the figure reported in an Australian study, where 20% of their participants increased alcohol purchase during the early pandemic period, although both are not directly comparable (31). Increased alcohol purchase during the pandemic may have been partly driven by more online alcohol marketing activities and availability of home alcohol delivery in some countries during lockdown (32, 33). This study found that those who stocked up on alcohol increased their alcohol drinking frequency. Similarly, a previous study found that those who often purchased alcohol online increased their quantity of alcohol consumption (33).

Several socioeconomic and environmental factors were associated with increased alcohol stock-up and use during lockdown. Psychological distress, working from home, ages 18–49 years, higher education achievement, and higher APC status were independently associated with increased alcohol stock-up and use. However, income loss was negatively associated with increased alcohol stock-up, and it was not independently associated with increased alcohol use. It could be that those who experienced income loss may not have been able to afford alcohol or may have been more conscious of their expenses. Living with children and being female appeared to have no effect on increased alcohol stock-up, despite these factors being strongly associated with increased alcohol use during lockdown. This could be because only 43.8% of all participants responded to the alcohol stock-up question or that women living with children prioritised stockpiling essential items over alcohol early in the pandemic.

Living alone was negatively associated with increased alcohol stock-up, and alcohol-use frequency did not increase among those living alone. Previous studies have found increased solitary drinking during the pandemic (31, 34–36). However, increased solitary drinking is not the same as increased alcohol use. People living alone may have moved from drinking with peers to solitary drinking during lockdown, without necessarily increasing their drinking frequency. The older age group, especially those living alone, was mentioned as a high-risk group in a few studies (15, 37–39). However, findings from the present study suggest that older people were unlikely to increase their alcohol use during the pandemic, which was also reported in several other studies (31, 33, 40, 41).

Similar to other studies during the pandemic (42–44), this study found that working from home was a risk factor for increased alcohol use during lockdowns. Two possible explanations were suggested. First, working from home may have enabled more free time at home, resulting in boredom, one of the most common reasons for drinking more alcohol during the pandemic (31, 45). Second, working from home might have increased stress because of the potential difficulties associated with working from home (46). Findings from the present study and the literature also indicate that working from home intersected with other factors associated with increased alcohol use, i.e., middle age, female gender, higher education attainment, living with children, and psychological distress.

Increased drinking among parents with children has been widely reported in the literature, and some studies reported that those with childcare responsibilities drank more frequently during the pandemic (28, 29, 35). It could be the case that, since most schools and childcare centres were closed during a lockdown and parents needed to supervise children while working from home, they faced increasing stress levels (29). Moreover, several studies found this to be the case specifically among women who often carry the burden of childcare within households (31, 46–48). Finally, it may be that those with higher educational achievement were able to afford to drink more during the pandemic because low-skilled workers, who often have lower academic achievement, were more likely to be affected economically due to lockdown.

Many studies have found a high level of mental health problems among people during the early phase of the pandemic (32, 40, 49–51). These studies also reported increased drinking among people with mental stress, a result supported by the present study. In previous studies, when people were asked why they increased drinking during the pandemic, anxiety or stress was one of the main reasons (31, 32, 45). Reasons for increased stress could be numerous during the pandemic, including uncertainty about the future and prolonged home confinement, apart from some factors mentioned above. However, it is important to notice that the direction of association between increased drinking and mental health symptoms is not always clear (41, 52), and it is sometimes mutually reinforcing (53). Previous studies have suggested that it was people prone to stress who mostly showed increased drinking during the pandemic as a coping behaviour (18, 34, 54, 55).

### Strengths and Limitations

There are several strong points of this study. Countries from all continents were included in the original data set, and data were collected in respective national languages. Still, although the sample of the original study was very large, the present study did not focus on individual differences between or within countries. Furthermore, the number of sampled participants in some countries was very small. Another limitation is the recruitment method of online self-selection and snowballing and the length of the survey, i.e., 30 min in average to complete. For example, the online nature of the study may have required higher levels of reading and digital proficiency, resulting in the overrepresentation of higher educated participants. There was also an overrepresentation of female participants, still, there were 8,539 (23%) male participants, which was sufficient for the statistical analysis for the alcohol use questions. The original study was primarily focused on cooking and diet, rather than alcohol use, and some variables were not ideal for the research question of this study. For example, it was not possible to estimate changes in the quantity, distinguish between types of alcohol, and other aspects of hazardous use of alcohol such as binge drinking were not captured in the data. Also, the alcohol stock-up question may have not addressed purchasing by other household members, and it did not offer information about the type of alcoholic beverage purchased or specific quantities. Additionally, respondents completed the survey at one point in time (cross-sectional), and so were recalling their frequency of consumption prior to the lockdown which was more than a month earlier. Finally, the period of data collection was close to the beginning of the pandemic and that may have been too early for people to fully experience the pandemic's impact.

### Implications for Health Care, Policies, and Research

As suggested in the literature, the present study further supports the importance of providing mental health support during the pandemic. Also, Helpline/Quitline and counselling services must be available and easy to access by all people throughout the pandemic and afterward. It is also important that ongoing support is available for people with alcohol use disorder during the pandemic. Previous research has found that family or professional support helped prevent relapse (17, 56). A previous study has suggested that people drinking more during lockdown are less likely to reduce their alcohol intake after lockdown (30). Particular attention should be given to vulnerable groups. For example, women living with children may require a special focus during the pandemic. Also, wellbeing support from employers may be vital for those working from home during a lockdown. Active screening for problematic alcohol use should be standard practise, so that timely healthcare and supports could be provided. Finally, it could prove helpful to formulate policies regulating alcohol retail and online marketing during the pandemic, so that alcohol-related harms could be minimised in the population (41, 57). Finally, further studies regarding the potential influence of region, culture, ethnicity, and religion in country-specific differences are required. Moreover, these studies should also pay attention to the influence of these factors within countries as well.

## Data Availability Statement

The authors confirm that the data supporting the findings of this study are available within the article and its Supplementary Materials. Please contact the authors to request access to the Corona Cooking Survey data.

## Ethics Statement

The studies involving human participants were reviewed and approved by the Ethics Committee for the Social Sciences and Humanities of the University of Antwerp approved the study protocol (approval code 20_46). The patients/participants provided their written informed consent to participate in this study.

## Author Contributions

CD, LT, PD, SP, KV, and IC conceived and designed the Corona Cooking Survey. SG led the collection of data in New Zealand and designed the sub-study on alcohol use with RR. SG, RR, and ZK contributed to interpretation of the findings. ZK wrote the initial draft, undertook all analyses, and with substantial input from RR. All authors revised the article and approved it for publication.

## Funding

This research was funded by the Research Foundation Flanders (G047518N) and Flanders Innovation and Entrepreneurship (HBC.2018.0397). These funding sources had no role in the design of the study, the analysis and interpretation of the data or the writing of, nor the decision to publish the manuscript.

## Conflict of Interest

The authors declare that the research was conducted in the absence of any commercial or financial relationships that could be construed as a potential conflict of interest.

## Publisher's Note

All claims expressed in this article are solely those of the authors and do not necessarily represent those of their affiliated organizations, or those of the publisher, the editors and the reviewers. Any product that may be evaluated in this article, or claim that may be made by its manufacturer, is not guaranteed or endorsed by the publisher.

## Footnotes

^1^One drink was referred to as a glass of an alcoholic beverage, including beer, wine or spirits.

^2^The questions: How much did you stock up on any of the foods/items below because of the Covid-19 pandemic? was used to get information about stockpiling, including stockpiling of alcoholic beverages before the lockdown.

^3^The present study included 37,206 participants for data cleaning. The main Corona Cooking research team used information of 37,207 participants (22). The difference of one participant is due to that one participant not answering the alcohol use question.

## References

[1] Rehm J Kilian C Ferreira-Borges C Jernigan D Monteiro M Parry CD. Alcohol use in times of the COVID 19: implications for monitoring and policy. Drug Alcohol Rev. (2020) 39:301–4. 10.1111/dar.13074 32358884 PMC7267161

[2] Mallet J Dubertret C Le Strat Y. Addictions in the COVID-19 era: current evidence, future perspectives a comprehensive review. Prog Neuro-Psychopharmacol Biol Psychiatry. (2020) 106:110070. 10.1016/j.pnpbp.2020.110070 32800868 PMC7420609

[3] Vetter S Rossegger A Rossler W Bisson JI Endrass J. Exposure to the tsunami disaster, PTSD symptoms and increased substance use–an Internet based survey of male and female residents of Switzerland. BMC Public Health. (2008) 8:1–6. 10.1186/1471-2458-8-92 18366682 PMC2277388

[4] Lau JT Yang X Pang E Tsui HY Wong E Wing YK. SARS-related perceptions in Hong Kong. Emerg Infect Dis. (2005) 11:417–24. 10.3201/eid1103.040675 15757557 PMC3298267

[5] Dubey MJ Ghosh R Chatterjee S Biswas P Chatterjee S Dubey S. COVID-19 and addiction. Diabetes Metab Syndr. (2020) 14:817–23. 10.1016/j.dsx.2020.06.008 32540735 PMC7282772

[6] Chick J. Alcohol and COVID-19. Alcohol Alcohol. (2020) 55:341–2. 10.1093/alcalc/agaa039 32400878 PMC7239251

[7] Balhara YPS Singh S Narang P. The effect of lockdown following COVID-19 pandemic on alcohol use and help seeking behaviour: observations and insights from a sample of alcohol use disorder patients under treatment from a tertiary care centre. Psychiatry Clin Neurosci. (2020) 74:440–1. 10.1111/pcn.13075 32463127 PMC7283852

[8] Wichaidit W Sittisombut M Assanangkornchai S Vichitkunakorn P. Self-reported drinking behaviors and observed violation of state-mandated social restriction and alcohol control measures during the COVID-19 pandemic: findings from nationally-representative surveys in Thailand. Drug Alcohol Depend. (2021) 221:108607. 10.1016/j.drugalcdep.2021.108607 33611025 PMC9759721

[9] De Jong M Vonk M Jenkins LS Reid S Reuter H. Prohibiting alcohol sales during the coronavirus disease 2019 pandemic has positive effects on health services in South Africa. Afr J Prim Health Care Fam Med. (2020) 12:1–4. 10.4102/phcfm.v12i1.2528 32787395 PMC7433289

[10] Reynolds J Wilkinson C. Accessibility of ‘essential' alcohol in the time of COVID-19: casting light on the blind spots of licensing? Drug Alcohol Rev. (2020) 39:305–8. 10.1111/dar.13076 32329548

[11] Martino F Brooks R Browne J Carah N Zorbas C Corben K. The nature and extent of online marketing by big food and big alcohol during the COVID-19 pandemic in Australia: content analysis study. JMIR Public Health Surveill. (2021) 7:e25202. 10.2196/25202 33709935 PMC7958974

[12] Nicholls E Conroy D. Possibilities and pitfalls? Moderate drinking and alcohol abstinence at home since the COVID-19 lockdown International. J Drug Policy. (2021) 88:103025. 10.1016/j.drugpo.2020.103025 33227638 PMC9759724

[13] Trangenstein PJ Curriero FC Webster D Jennings JM Latkin C Eck R. Outlet type, access to alcohol, and violent crime. Alcohol Clin Exp Res. (2018) 42:2234–45. 10.1111/acer.13880 30256427 PMC6214776

[14] Testino G. Are patients with alcohol use disorders at increased risk for Covid-19 infection? Alcohol Alcohol. (2020) 55:344–6. 10.1093/alcalc/agaa037 32400858 PMC7239257

[15] Satre DD Hirschtritt ME Silverberg MJ Sterling SA. Addressing problems with alcohol and other substances among older adults during the COVID-19 pandemic. Am J Geriatr Psychiatry. (2020) 28:780–3. 10.1016/j.jagp.2020.04.012 32359882 PMC7174977

[16] Farhoudian A Baldacchino A Clark N Gerra G Ekhtiari H Dom G. COVID-19 and substance use disorders: recommendations to a comprehensive healthcare response. An international society of addiction medicine practice and policy interest group position paper. Basic Clin Neurosci. (2020) 11:133. 10.32598/bcn.11.covid19.1 32855772 PMC7368103

[17] Kim JU Majid A Judge R Crook P Nathwani R Selvapatt N. Effect of COVID-19 lockdown on alcohol consumption in patients with pre-existing alcohol use disorder. Lancet Gastroenterol Hepatol. (2020) 5:886–7. 10.1016/S2468-1253(20)30251-X 32763197 PMC7403133

[18] Chodkiewicz J Talarowska M Miniszewska J Nawrocka N Bilinski P. Alcohol consumption reported during the COVID-19 pandemic: the initial stage. Int J Environ Res Public Health. (2020) 17:4677. 10.3390/ijerph17134677 32610613 PMC7369979

[19] Capasso A Jones AM Ali SH Foreman J Tozan Y DiClemente RJ. Increased alcohol use during the COVID-19 pandemic: the effect of mental health and age in a cross-sectional sample of social media users in the US. Prev Med. (2021) 145:106422. 10.1016/j.ypmed.2021.106422 33422577 PMC9063034

[20] Kilian C Rehm J Allebeck P Braddick F Gual A Barták M. Alcohol consumption during the COVID-19 pandemic in Europe: a large-scale cross-sectional study in 21 countries. Addiction. (2021) 116:3369–80. 10.21203/rs.3.rs-148341/v2 34109685

[21] Bakaloudi DR Jeyakumar DT Jayawardena R Chourdakis M. The impact of COVID-19 lockdown on snacking habits, fast-food and alcohol consumption: a systematic review of the evidence. Clinical Nutrition. (2021). 10.1016/j.clnu.2021.04.020 34049747 PMC8052604

[22] De Backer C Teunissen L Cuykx I Decorte P Pabian S Gerritsen S. An Evaluation of the COVID-19 pandemic and perceived social distancing policies in relation to planning, selecting, and preparing healthy meals: an observational study in 38 Countries worldwide. Front Nutr. (2020) 7:621726. 10.3389/fnut.2020.621726 33614693 PMC7890074

[23] University of Oxford. Covid-19 Government Response Tracker. University of Oxford, Oxford: Blavatnik School of Government (2021).

[24] World Health Organisation. Alcohol, total per capita (15+ years) consumption (in litres of pure alcohol). World Health Organisation, Geneva: World Health Organization (2021).

[25] World Health Organization. Global status report on alcohol and health 2018. World Health Organization, Geneva: World Health Organization (2019).

[26] Kessler RC Andrews G Colpe LJ Hiripi E Mroczek DK Normand SL. Short screening scales to monitor population prevalences and trends in non-specific psychological distress. Psychol Med. (2002) 32:959–76. 10.1017/S0033291702006074 12214795

[27] Krynen A Osborne D Duck IM Houkamau CA Sibley CG. Measuring psychological distress in New Zealand: item response properties and demographic differences in the Kessler-6 screening measure. NZ J Psychol. (2013) 42:69–83.

[28] MacMillan T Corrigan MJ Coffey K Tronnier CD Wang D Krase K. Exploring factors associated with alcohol and/or substance use during the COVID-19 pandemic. Int J Ment Health Addict. (2021) 1–10. 10.1007/s11469-020-00482-y 33519318 PMC7837073

[29] Boschuetz N Cheng S Mei L Loy VM. Changes in alcohol use patterns in the United States During COVID-19 pandemic. WMJ. (2020) 171–6. 33091284

[30] Alcohol Change UK. New Research Reveals that Without Action Lockdown Drinking Habits May be Here to Stay. In press (2020).

[31] Foundation for Alcohol Research and Education. Alcohol use and harm during Covid-19. In press, Canberra: Foundation for Alcohol Research & Education (2020).

[32] Health Promotion Agency. The Impact of Lockdown on Health Risks Behaviours. In press, Wellington: Health Promotion Agency (2020).

[33] Huckle T Parker K Romeo JS Casswell S. Online alcohol delivery is associated with heavier drinking during the first New Zealand COVID-19 pandemic restrictions. Drug Alcohol Rev. (2020) 40:826–34. 10.1111/dar.13222 33283442 PMC7753625

[34] McPhee MD Keough MT Rundle S Heath LM Wardell JD Hendershot CS. Depression, environmental reward, coping motives and alcohol consumption during the COVID-19 pandemic. Front Psychiatry. (2020) 11:1128. 10.3389/fpsyt.2020.574676 33192708 PMC7661794

[35] Wardell JD Kempe T Rapinda KK Single A Bilevicius E Frohlich JR. Drinking to cope during COVID-19 pandemic: the role of external and internal factors in coping motive pathways to alcohol use, solitary drinking, and alcohol problems. Alcohol Clin Exp Res. (2020) 44:2073–83. 10.1111/acer.14425 32870516

[36] Dumas TM Ellis W Litt DM. What does adolescent substance use look like during the COVID-19 pandemic? Examining changes in frequency, social contexts, and pandemic-related predictors. J Adoles Health. (2020) 67:354–61. 10.1016/j.jadohealth.2020.06.018 32693983 PMC7368647

[37] Villanueva VJ Motos P Isorna M Villanueva V Blay P Vázquez-Martínez A. Impact of confinement measures on the Covid-19 pandemic on alcohol risk consumption. Rev Esp Salud Publica. (2021) 95:e202101015. 33468986

[38] Blazer DG Wu L. The epidemiology of substance use and disorders among middle aged and elderly community adults: national survey on drug use and health. Am J Geriatr Psychiatry. (2009) 17:237–45. 10.1097/JGP.0b013e318190b8ef 19454850 PMC2721326

[39] Nordeck CD Riehm KE Smail EJ Holingue C Kane JC Johnson RM. Changes in drinking days among US adults during the COVID-19 pandemic. Addiction. (2021). 10.1111/add.15622 34159674 PMC8441933

[40] Ahmed MZ Ahmed O Aibao Z Hanbin S Siyu L Ahmad A. Epidemic of COVID-19 in China and associated psychological problems. Asian J Psychiatr. (2020) 51:102092. 10.1016/j.ajp.2020.102092 32315963 PMC7194662

[41] Tran TD Hammarberg K Kirkman M Nguyen HTM Fisher J. Alcohol use and mental health status during the first months of COVID-19 pandemic in Australia. J Affect Disord. (2020) 277:810–3. 10.1016/j.jad.2020.09.012 33065821 PMC7476559

[42] Szajnoga D Klimek-Tulwin M Piekut A. COVID-19 lockdown leads to changes in alcohol consumption patterns. Results from the Polish national survey. J Addict Dis. (2020) 1–12. 10.1080/10550887.2020.1848247 33308059

[43] Alpers SE Skogen JC Mæland S Pallesen S Rabben ÅK Lunde L. Alcohol consumption during a pandemic lockdown period and change in alcohol consumption related to worries and pandemic measures. Int J Environ Res Public Health. (2021) 18:1220. 10.3390/ijerph18031220 33572994 PMC7908087

[44] Schmits E Glowacz F. Changes in alcohol use during the COVID-19 pandemic: impact of the lockdown conditions and mental health factors. Int J Ment Health Addict. (2021) 1–12. 10.1007/s11469-020-00432-8 33424513 PMC7781407

[45] Grossman ER Benjamin-Neelon S Sonnenschein S. Alcohol consumption during the COVID-19 pandemic: a cross-sectional survey of US adults. Int J Environ Res Public Health. (2020) 17:9189. 10.3390/ijerph17249189 33316978 PMC7763183

[46] Neill E Meyer D Toh WL van Rheenen TE Phillipou A Tan EJ. Alcohol use in Australia during the early days of the COVID-19 pandemic: initial results from the COLLATE project. Psychiatry Clin Neurosci. (2020) 74:542–9. 10.1111/pcn.13099 32602150 PMC7436134

[47] Calarco JM Meanwell EV Anderson E Knopf A. “My husband thinks I'm crazy”: COVID-19-related conflict in couples with young children. SocArXiv. (2020). 10.31235/osf.io/cpkj6

[48] Calarco JM Anderson EM Meanwell EV Knopf A. “Let's not pretend It's fun”: How COVID-19-related school and childcare closures are damaging mothers' well-being. SocArXiv. (2020). 10.31235/osf.io/jyvk4

[49] Panno A Carbone GA Massullo C Farina B Imperatori C. COVID-19 related distress is associated with alcohol problems, social media and food addiction symptoms: insights From the Italian experience during the lockdown. Front Psychiatry. (2020) 11:1314. 10.3389/fpsyt.2020.577135 33324256 PMC7723899

[50] Stanton R To QG Khalesi S Williams SL Alley SJ Thwaite TL. Depression, anxiety and stress during COVID-19: associations with changes in physical activity, sleep, tobacco and alcohol use in Australian adults. Int J Environ Res Public Health. (2020) 17:4065. 10.3390/ijerph17114065 32517294 PMC7312903

[51] Wang Y Lu H Hu M Wu S Chen J Wang L. Alcohol consumption in China before and during CoViD-19: preliminary results from an online retrospective survey. Front Psychiatry. (2020) 11:597826. 10.3389/fpsyt.2020.597826 33324263 PMC7723925

[52] Jacob L Smith L Armstrong NC Yakkundi A Barnett Y Butler L. Alcohol use and mental health during COVID-19 lockdown: a cross-sectional study in a sample of UK adults. Drug Alcohol Depend. (2021) 219:108488. 10.1016/j.drugalcdep.2020.108488 33383352 PMC7768217

[53] Lechner WV Laurene KR Patel S Anderson M Grega C Kenne DR. Changes in alcohol use as a function of psychological distress and social support following COVID-19 related university closings. Addict Behav. (2020) 110:106527. 10.1016/j.addbeh.2020.106527 32679435 PMC7319610

[54] Koopmann A Georgiadou E Kiefer F Hillemacher T. Did the general population in Germany drink more alcohol during the COVID-19 pandemic lockdown? Alcohol and Alcoholism. (2020) 55:698–9. 10.1093/alcalc/agaa058 32556079 PMC7337704

[55] Sallie SN Ritou V Bowden-Jones H Voon V. Assessing international alcohol consumption patterns during isolation from the COVID-19 pandemic using an online survey: highlighting negative emotionality mechanisms. BMJ Open. (2020) 10:e044276. 10.1136/bmjopen-2020-044276 33243820 PMC7692002

[56] Yazdi K Fuchs-Leitner I Rosenleitner J Gerstgrasser NW. Impact of the COVID-19 pandemic on patients with alcohol use disorder and associated risk factors for relapse. Front Psychiatry. (2020) 11. 10.3389/fpsyt.2020.620612 33391060 PMC7772314

[57] McKetta S Morrison CN Keyes KM. Trends in US alcohol consumption frequency during the first wave of the SARS-CoV-2 pandemic. Alcohol Clin Exp Res. (2020) 45:773–83. 10.1111/acer.14575 33587290 PMC8014717

