# Supplementary material for: Socioeconomic and Environmental Factors Associated With Increased Alcohol Purchase and Consumption in 38 Countries During the Covid-19 Pandemic
Source: Front Psychiatry. 2022 Jan 14;12:802037. doi: 10.3389/fpsyt.2021.802037 (PMC8795628; doi:10.3389/fpsyt.2021.802037)
Supplement: Supplementary file 1 [file Data_Sheet_1.docx]

Table S1: Country list with the Participants’ Gender

| **Country of residence** | **Female** | **Male** | **Total (n, %)** |
| --- | --- | --- | --- |
| Australia | 464 | 33 | 497 (1.3) |
| Austria | 302 | 65 | 367 (1.0) |
| Arab countries | 11,006 | 2,717 | 13,723 (36.9) |
| Belgium | 5,623 | 1,447 | 7,070 (19.0) |
| Brazil | 397 | 151 | 548 (1.5) |
| Canada | 656 | 162 | 818 (2.2) |
| Chile | 591 | 274 | 865 (2.3) |
| China | 202 | 329 | 531 (1.4) |
| Denmark | 397 | 427 | 824 (2.2) |
| Ecuador | 501 | 295 | 796 (2.1) |
| Finland | 751 | 64 | 815 (2.2) |
| France | 136 | 54 | 190 (0.5) |
| Germany | 339 | 267 | 606 (1.6) |
| Greece | 534 | 275 | 809 (2.2) |
| Ireland | 373 | 121 | 494 (1.3) |
| Italy | 267 | 60 | 327 (0.9) |
| Japan | 298 | 278 | 576 (1.5) |
| Mexico | 461 | 160 | 621 (1.6) |
| Netherlands | 622 | 123 | 745 (2.0) |
| New Zealand | 2,676 | 314 | 2,990 (8.0) |
| Peru | 456 | 139 | 595 (1.6) |
| Poland | 290 | 247 | 537 (1.4) |
| Romania | 261 | 69 | 330 (0.9) |
| Singapore | 75 | 42 | 117 (0.3) |
| South Africa | 108 | 20 | 128 (0.3) |
| Spain | 557 | 178 | 735 (2.0) |
| Uganda | 161 | 160 | 321 (0.9) |
| United Kingdom | 75 | 46 | 121 (0.3) |
| United States | 88 | 22 | 110 (0.3) |
| **Total (n, %)** | 28667 (77.0) | 8539 (23.0) | 37206 (100.0) |

Table S2: Effect of distress on increased alcohol stock-up, adjusted for other factors

| **Variable** | **Category** | **Odds Ratio** | **95% CI** | **P-value** |
| --- | --- | --- | --- | --- |
| Psychological distress | Mild | Reference | | |
|  | Moderate | 1.41 | 1.30-1.53 | <0.001 |
|  | Severe | 1.89 | 1.71-2.09 | <0.001 |
| Household structure | Adult only | Reference | | |
|  | Includes child | 0.94 | 0.86-1.02 | 0.109 |
|  | Alone | 0.86 | 0.77-0.95 | 0.005 |
| Working status | Working at workplace | 1.08 | 0.95-1.22 | 0.235 |
|  | Working at home | 1.53 | 1.39-1.69 | <0.001 |
|  | Unemployed | Reference | | |
|  | Student | 0.87 | 0.73-1.03 | 0.106 |
| Income loss | No | Reference | | |
|  | Yes | 0.84 | 0.77-0.91 | <0.001 |
| Gender | Male | Reference | | |
|  | Female | 0.98 | 0.90-1.07 | 0.696 |
| Age | 18-25 | 1.45 | 1.26-1.66 | <0.001 |
|  | 26-49 | 1.42 | 1.3-1.56 | <0.001 |
|  | >49 | Reference | | |
| Education | High school | Reference | | |
|  | Bachelor | 1.22 | 1.10-1.34 | <0.001 |
|  | Postgraduate | 1.17 | 1.05-1.30 | 0.003 |
| Country based on alcohol use status | Low and medium APC (<7.5L) | Reference | | |
|  | High and very high APC (≥7.5L) | 1.16 | 1.02-1.32 | 0.024 |

Table S3: Effect of distress on increased alcohol use, adjusted for other factors

| **Variable** |  | **Category** | **Odds Ratio** | **95% CI** | **P-value** |
| --- | --- | --- | --- | --- | --- |
| Psychological distress | Mild | | Reference | | |
|  | Moderate | | 1.23 | 1.16 - 1.30 | <0.001 |
|  | Severe | | 1.40 | 1.30 - 1.50 | <0.001 |
| Household structure | Adult only | | Reference | | |
|  | Includes child | | 1.18 | 1.11 - 1.24 | <0.001 |
|  | Alone | | 0.87 | 0.79 - 0.97 | 0.009 |
| Working status | Working at workplace | | 1.08 | 0.99 - 1.17 | 0.098 |
|  | Working at home | | 1.36 | 1.27 - 1.46 | <0.001 |
|  | Unemployed | | Reference | | |
|  | Student | | 0.94 | 0.86 - 1.04 | 0.232 |
| Gender | Male | | Reference | | |
|  | Female | | 1.11 | 1.04 - 1.19 | 0.001 |
| Age | 18-25 | | 1.40 | 1.28 - 1.54 | <0.001 |
|  | 26-49 | | 1.34 | 1.24 - 1.44 | <0.001 |
|  | >49 | | Reference | | |
| Education | High school | | Reference | | |
|  | Bachelor | | 1.19 | 1.11 - 1.27 | <0.001 |
|  | Postgraduate | | 1.18 | 1.09 - 1.28 | <0.001 |
| Country based on alcohol use status | Low and medium APC (<7.5L) | | Reference | | |
|  | High and very high APC (≥7.5L) | | 1.41 | 1.33 - 1.50 | <0.001 |
